# Risk Factors for Early- and Late-Onset Cholecystitis after Y-Configured Metal Stent Placement in Patients with Malignant Hilar Biliary Obstruction: A Single-Center Study

**DOI:** 10.3390/jcm12134354

**Published:** 2023-06-28

**Authors:** Jonghyun Lee, Sung Yong Han, Donghoon Baek, Gwang Ha Kim, Geun Am Song, Dong Uk Kim

**Affiliations:** 1 Department of Internal Medicine, Pusan National University Hospital, Busan 49241, Republic of Korea; keiasikr@nate.com (J.L.); mirsaint@hanmail.net (S.Y.H.); dhbeak77@gmail.com (D.B.); doc0224@chol.com (G.H.K.); gasong@pusan.ac.kr (G.A.S.); 2 Biomedical Research Institute, Pusan National University Hospital, Busan 49241, Republic of Korea

**Keywords:** cholecystitis, self-expandable metallic stents, bile duct neoplasm

## Abstract

This study evaluated the prevalence and risk factors of early- (within 7 days of placement) and late-onset (after 7 days of placement) cholecystitis after Y-configured metal stent placement. Between June 2005 and August 2020, 109 patients who had been treated with Y-configured metal stents for malignant hilar obstruction were enrolled in the study. We retrospectively analyzed the potential risk factors for post-stent cholecystitis. The presence of diabetes (*p* = 0.042), the length of the common part of the Y-stent (*p* = 0.017), filling of the gallbladder with contrast medium during the procedure (*p* = 0.040), and tumor invasion of the cystic duct accompanied by filling the gallbladder with contrast medium during metal stent placement (*p* = 0.001) were identified as important risk factors. In cases of late-onset cholecystitis, stent obstruction (*p* = 0.004) and repeated endoscopic procedures due to stent malfunction (*p* = 0.024) were significant risk factors. In the multivariate logistic regression analysis, significant risk factors were the length of the common part of the Y-stent (*p* = 0.032) in early-onset cholecystitis and stent obstruction (*p* = 0.007) in late-onset cholecystitis. This study demonstrated that early-onset cholecystitis may occur in patients according to the length of the common portion of the Y-stent. In contrast, late-onset cholecystitis may occur in patients with stent obstruction.

## 1. Introduction

Malignant hilar obstruction can be caused by various types of tumors that obstruct the bile duct via direct expansion and lymphatic metastasis [1]. The average 5–year survival rate of patients with malignant hilar obstruction is approximately 10% [2]. If a patient develops obstructive jaundice, biliary drainage should be performed to prevent cholangitis, biliary sepsis, and hepatic failure [3]. Therefore, it is vital to consider nonsurgical treatment in patients with a malignant hilar obstruction that cannot be treated surgically.

The endoscopic insertion of metal stents is a well-established and widely used palliative treatment for obstructive jaundice resulting from unresectable malignant hilar obstruction. The use of plastic stents to relieve malignant hilar obstruction has shown poorer results than the use of metal stents in some studies [4]. In cases of hilar obstruction, a relatively long stent is generally used. Plastic stents have narrower lumens than metal stents; therefore, plastic stents are more likely to become blocked. In situations where both biliary branches are blocked in patients with malignant hilar obstruction, it is essential to manage both biliary tracts effectively. In such scenarios, a Y-shaped metal stent could be used to manage the occlusion [5,6,7,8]. To ensure optimal placement, the first stent is typically placed endoscopically in a duct that is difficult to access. The left stent is placed before the right stent, due to its higher level of difficulty in terms of access. However, this may vary depending on the specific circumstances.

**Figure 1:**
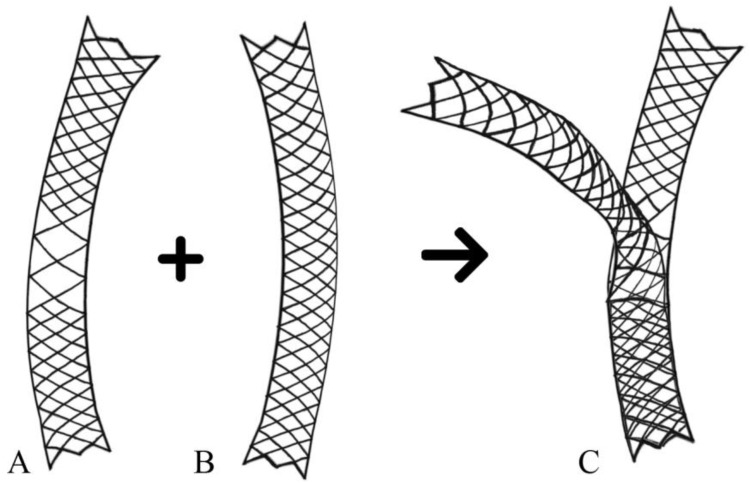
An illustration of the Y-shaped metal stent used in the ‘through-the-mesh’ method. (**A**) Initial stent with a wider mesh opening in the middle part. (**B**) Second stent. (**C**) Combination with the second stent through the wider mesh portion of the first stent.

A Y-shaped metal stent is generally used in the ‘through-the-mesh’ (TTM) stent procedure. This procedure allows the second stent to pass through the open mesh wall of the first stent and enter the opposite side of the duct. Studies have reported the use of a specific SEMS with a wide mesh opening in the middle [9]. Combined with the second stent, the wider mesh portion of the first stent produces a Y-shaped array (Figure 1). In general, the Y-stent is located in the main right and left intrahepatic bile ducts. Nevertheless, placing a stent in the right anterior and posterior ducts poses challenges due to their relatively narrow structure. However, it is worth noting that if an obstruction increases the diameters of both ducts, a Y-stent can be used.

Recently, technical advancements in therapeutic endoscopic retrograde cholangiopancreatography (ERCP) and the development of various metal stents have reduced procedure-related complications such as cholangitis, pancreatitis, cholecystitis, haemobilia, and stent migration. At the same time, advances in the treatment of malignancies have extended the overall patient survival period. As the survival time of patients increases, late complications after the metal stent procedure, such as stent occlusion, recurrent cholangitis, liver abscesses, and cholecystitis, will also increase. Therefore, despite advances in technology, patients with malignant hilar obstruction still experience poor outcomes and complications related to palliative endoscopic treatment.

Despite advancements in the management of hilar obstruction using metal stents, complications such as cholecystitis can still arise, with incidence rates ranging from 1.9% to 12% after metal stent placement [10,11]. The reason for the cholecystitis is expected to be related to environmental changes after metal stent placement. One contributing factor is the reflux of duodenal contents after endoscopic sphincterotomy, which can lead to bacterial colonization of the biliary system. Additionally, the presence of metal stents can exert pressure on the tumor mass, causing progressive obstruction of the cystic duct and gallbladder dysfunction. Although these factors can be predicted, the risk factors for cholecystitis after metal stent placement have not yet been established.

Therefore, it is clinically meaningful to investigate the risk factors associated with the development of early- and late-onset cholecystitis after metal stent implantation. This study aimed to determine the risk factors for early- and late-onset cholecystitis in patients with malignant hilar obstruction who underwent endoscopic Y-shaped metal stent implantation.

## 2. Materials and Methods

### 2.1. Patients

**Figure 2:**
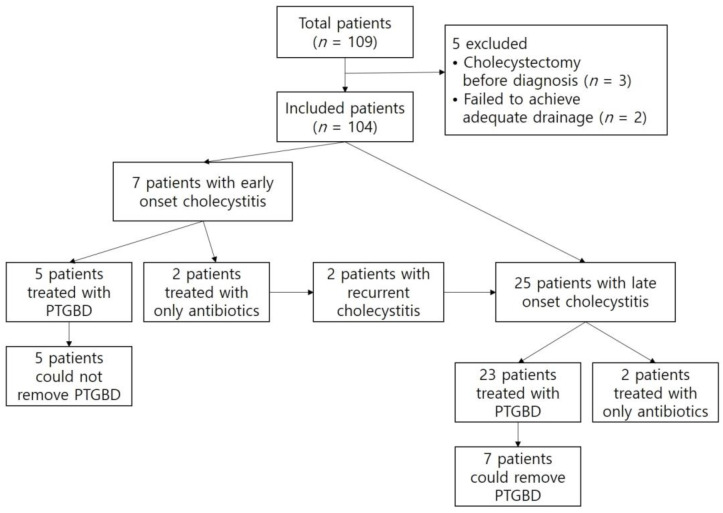
A flow chart of the patients who were involved in this study. PTGBD, Percutaneous Transhepatic gallbladder drainage.

From June 2005 to December 2020, we retrospectively reviewed the data of all patients who underwent therapeutic ERCP for the palliation of malignant hilar obstruction at a single tertiary referral hospital. The study was conducted in accordance with the Declaration of Helsinki and was approved by the Institutional Review Board (or Ethics Committee of the Institutional Review Board) of Pusan National University Hospital (2305-017-127, 26 May 2023). The inclusion criteria were as follows: unresectable malignant hilar obstruction, technical success of bilateral Y-configured stent placement, including selective IHD cannulation, and no evidence of cholecystitis at baseline. The exclusion criteria were a history of cholecystectomy before ERCP and unresolved bile duct obstruction with a bilateral Y-configured stent. A total of 109 patients were found to have Y-configured metal stents. Of these, 3 patients were excluded due to cholecystectomy before diagnosis, while adequate drainage could not be achieved in 2 patients, requiring them to undergo percutaneous biliary drainage. Finally, a total of 104 patients were enrolled in this study (Figure 2).

### 2.2. Methods

**Figure 3:**
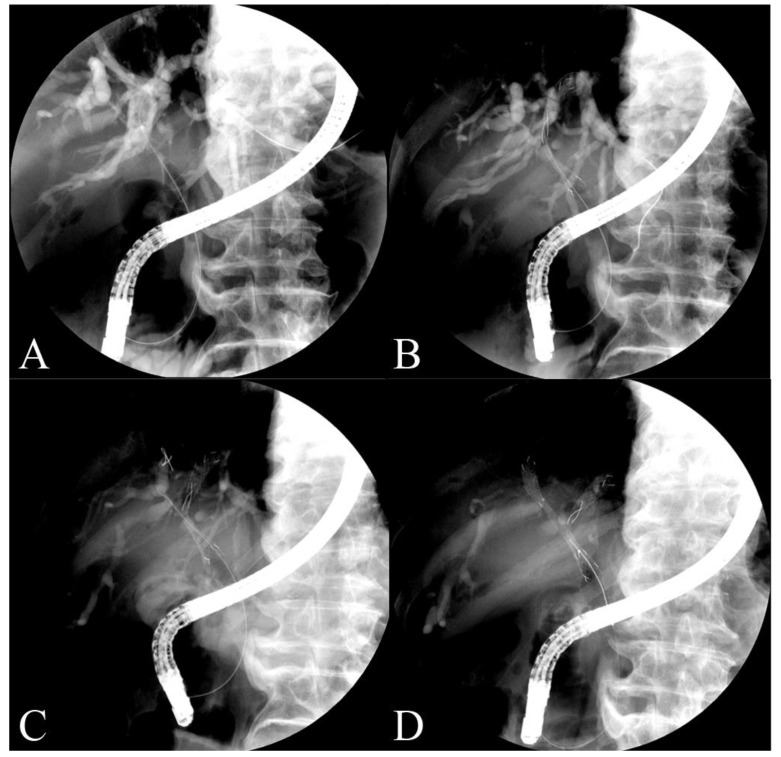
Images from malignant hilar obstruction patients who underwent the Y-stent procedure. (**A**) The guide wire is located in the left intrahepatic bile duct. (**B**) The metal stent with a wide mesh in the middle is placed. (**C**) A second guide wire is located in the right intrahepatic bile duct through the wide area. (**D**) The second stent is located in the right intrahepatic bile duct across the wide area of the first stent through the guide wire.

An experienced endoscopist placed the endoscopic bilateral Y-shaped metal stent (Y-type biliary Niti-S stent; Taewoong Inc., Seoul, Republic of Korea). Using a guidewire, the first stent was endoscopically inserted into the intrahepatic bile duct across the hilar obstruction. The second biliary stent was placed via a guidewire through a wide mesh area in the middle of the first stent in the opposite intrahepatic bile duct. The wider opening of the first Y-stent allowed for the easy passage and wide extension of the second stent guidewire. Stents, when deployed, form a Y-shaped configuration that enables the simultaneous treatment of both biliary branches affected by the obstruction (Figure 3).

All patients received intravenous antibiotics before and 3 days after the procedure. In cases where signs of cholecystitis, cholangitis, or other infections were present, the administration of antibiotic treatment was extended until the symptoms were under control. The definition of cholecystitis used was based on the Tokyo Guidelines (2018), which consider systemic signs of inflammation (such as fever, elevated white blood cell count, or elevated C-reactive protein level) as well as the imaging findings of cholecystitis, accompanied by local signs of inflammation (such as a recently developed positive Murphy’s sign, right upper quadrant pain, mass, or tenderness). Early-onset cholecystitis was defined as cholecystitis occurring within 7 days of stent implantation. If the disease occurred 7 days after stent placement, it was defined as late-onset cholecystitis. Patients with early-onset and recurrent cholecystitis were also included among the patients with late-onset cholecystitis.

Antibiotics were administered when a patient was diagnosed with cholecystitis. Percutaneous transhepatic gallbladder drainage (PTGBD) was performed when symptoms worsened rapidly or did not improve within 3 days of antibiotic administration. Potential risk factors, including patient-related factors such as sex, age, diabetes mellitus, gallstones, pre-ERCP cholangitis and procedure-related factors such as filling the gallbladder with contrast medium during the endoscopic procedure, tumor invasion of the cystic duct, and repeated endoscopic procedures due to stent malfunction, were retrospectively analyzed using the hospital database.

This study spanned a total of 15 years. Considering the long duration, it was divided into 2 distinct periods: previous groups (2005–2012) and recent groups (2013–2020). The division into these groups enhanced the accuracy of the analysis by allowing for the comparison of risk factors associated with early- and late-onset cholecystitis in each group.

### 2.3. Statistical Analysis

Independent sample *t*-tests were used to compare the continuous variables. Data are expressed as the mean (SD). Some patients underwent more than one ERCP procedure. For the data analysis, factors from different ERCP procedures were assumed to be statistically independent. The correlation between cholecystitis and the potential risk factors was assessed using univariate analysis. Multivariate analysis was performed to identify the meaningful factors. Data were analyzed using the statistical program IBM SPSS Statistics for Windows 10, version 22 (IBM Corp., Armonk, NY, USA). Statistical significance was set at *p* < 0.05 for all tests.

## 3. Results

Of the 104 patients, 55 were male, and 40 were female, with a mean age of 72.1 ± 10.8 years. The causes of malignant hilar obstruction were cholangiocarcinoma in 77 (74.0%) patients, gallbladder cancer in 25 patients (24.0%), hepatocellular carcinoma in 1 patient (1%), and other cancers in 1 patient (1%).

Thirty patients (28.8%) were diagnosed with cholecystitis after metal stent placement. Early- and late-onset cholecystitis occurred in 7 (6.7%) and 25 (24.0%) patients, respectively (Table 1 and Table 2). Two patients with early cholecystitis who experienced recurrent cholecystitis were also included in the number of patients with late cholecystitis.

**Figure 4:**
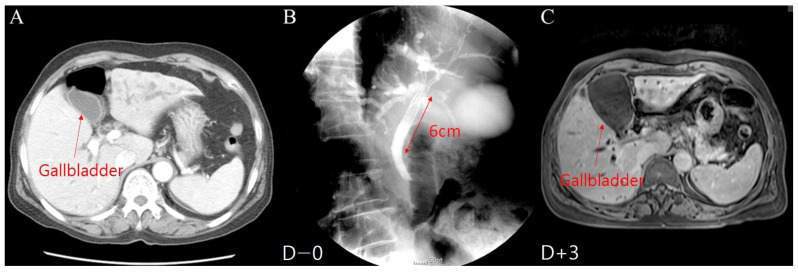
Early-onset cholecystitis in patients who underwent a Y-stent procedure with a long (6 mm) common part: (**A**) Gallbladder before the Y-stent procedure. (**B**) Y-stent placement. (**C**) Early-onset cholecystitis after the Y-stent procedure.

The seven patients with early-onset cholecystitis included three males and four females, aged 62.7 ± 4.9 years. The average time to early-onset cholecystitis was 3.1 days (1–6 days) after metal stent placement (Figure 4). The causes of malignant hilar obstruction were cholangiocarcinoma in five patients and gallbladder cancer in two patients, accompanied by cystic duct invasion of the malignant tumor in six cases, as shown by an abdominal CT scan. The length of the common part of the Y-stent was 5.4 ± 0.8 cm, and the stent was patent for 120.7 ± 157.2 days in early-onset cholecystitis patients. Of these patients, five (71.4%) underwent gallbladder filling with contrast medium during metal stent placement. Four patients (57.1%) had diabetes mellitus. Five patients with early-onset cholecystitis were treated with percutaneous cholecystostomy shortly after diagnosis and the PTGBD catheter could not be removed. Two patients treated with antibiotic administration experienced a relapse of cholecystitis. The patients survived for 176.3 days on average (10–442 days).

The significant risk factors for the development of early-onset cholecystitis were the length of the common part of the Y-stent (*p* = 0.017), the presence of diabetes mellitus (*p* = 0.042), filling the gallbladder with contrast medium during metal stent placement (*p* = 0.040), and tumor invasion of the cystic duct accompanied by filling the gallbladder with contrast medium during metal stent placement (*p* = 0.001) (Table 1). There were no significant differences in sex, age, pre-ERCP cholangitis, gallbladder (GB) stones, or initial laboratory findings between the early-onset cholecystitis and non-cholecystitis groups. In the multivariate logistic regression analysis, the length of the common part of the Y-stent (*p* = 0.032; odds ratio (OR) 3.323 (1.111–9.941)) was a significant risk factor for early-onset cholecystitis (Table 1).

**Figure 5:**
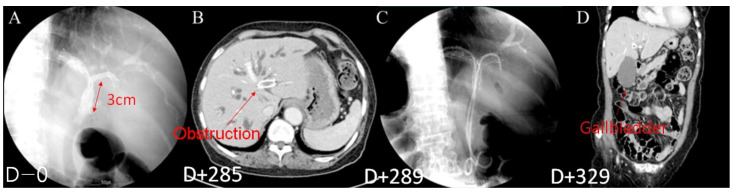
Late-onset cholecystitis in patients with Y-stent obstruction; (**A**) Y-stent placement (Day 0). (**B**) Stent obstruction (Day 285). (**C**) Placement of ERBD in an obstructed stent (Day 289). (**D**) Late-onset cholecystitis (Day 329).

The 25 patients with late-onset cholecystitis included 10 males and 15 females, aged 71.4 ± 8.5 years. Late-onset cholecystitis occurred at an average interval of 198 days (range: 17–552 days) after endoscopic metal stent insertion (Figure 5). The causes of the obstruction were gallbladder cancer in 3 patients and cholangiocarcinoma in 22 patients. The length of the common part of the Y-stent was 7.3 ± 0.9 cm, and the stent was patent for 256.4 ± 177.1 days in late-onset cholecystitis patients. Of these patients, 21 (84%) experienced stent obstruction due to tumor ingrowth, overgrowth, sludge, stones, and repeated procedures, such as percutaneous transhepatic biliary drainage (PTBD) and endoscopic retrograde biliary drainage (ERBD). The average survival time of patients with late-onset cholecystitis was 385 days (range: 31–1030 days) after metal stent placement. The stents were patent for 261.2 ± 185.2 days. Twenty-three patients with late-onset cholecystitis were treated with percutaneous cholecystostomy after diagnosis, and seven of them were able to remove the PTGBD. The mean duration of PTGBD was 90.7 ± 35.9 days.

The significant risk factors for the development of late-onset cholecystitis were stent obstruction (*p* = 0.004) and a repeat procedure owing to stent obstruction (*p* = 0.001) (Table 2). There were no significant differences in sex, age, length of the common part of the Y-stent, stent patent days, survival days, pre-ERCP cholangitis, previous early-onset cholecystitis, GB stones, presence of diabetes, GB filling with contrast medium, invasion of the cystic duct, or initial laboratory findings between the late-onset cholecystitis and non-cholecystitis groups. In multivariate logistic regression analysis, stent obstruction (*p* = 0.007, OR 5.879 (1.641–21.059)) was an important risk factor for late-onset cholecystitis (Table 2).

The previous group (2005–2012) comprised 90 patients. Of these, 7 patients developed early-onset cholecystitis, and 24 developed late-onset cholecystitis. The risk factors in the early-onset cholecystitis group were diabetes (*p* = 0.046), GB filling with contrast medium and cystic duct invasion (*p* = 0.002), and the length of the common part of the Y-stent (*p* = 0.019). The risk factors in the late-onset cholecystitis group were cholangitis before ERCP (*p* = 0.027), GB filling with contrast medium and cystic duct invasion (*p* = 0.025), stent obstruction (*p* = 0.004), and repeated procedures (*p* = 0.001) (Table 3).

In the more recent group (2013–2020), the Y-stent was used in 14 patients. None of the patients had early-onset cholecystitis, and one patient developed late-onset cholecystitis. In the late-onset cholecystitis group, the risk factors were diabetes (*p* = 0.047), stent obstruction (*p* = 0.047), and procedure repetition (*p* = 0.047) (Table 4).

## 4. Discussion

Malignant hilar obstruction frequently occurs because of cholangiocarcinoma, gallbladder cancer, and metastatic cancer, along with the direct or indirect invasion of the hepatic confluence [12,13]. To manage the obstruction, a metal stent can be a good option. However, complication rates of 15–34% have been reported in several case series [14,15,16,17,18,19]. Stent-related cholecystitis has been reported in 1.9% [10] to 12% [11] of metal stent insertion cases in previous studies. In the present study, the incidence of early-onset cholecystitis was 6.7%. Although this study was based on a stent-in-stent procedure, it was similar to a single-stent study involving patients with malignant obstructive disease. However, 30 (28.8%) patients were diagnosed with cholecystitis when early- and late-onset cholecystitis were combined. According to a recent study, prophylactic endoscopic ultrasound (EUS)-guided gallbladder drainage reduces acute cholecystitis [20]. In the study, 22 patients were included in each group. Five patients in the control group developed cholecystitis (22.7%); conversely, none developed cholecystitis in the experimental group. As the number of patients in this study was small, further studies are required. However, based on the results of this study, performing preventive EUS in patients with risk factors for cholecystitis may be helpful.

Filling the gallbladder with a contrast medium is the sole risk factor for early-onset cholecystitis. This seems likely to be due to the toxicity of the contrast medium to the gallbladder epithelial cells [21] and contamination of the sterilized contrast medium following its passage through the biliary tract. Minimal manipulation during stent placement, the injection of sterile contrast medium, complete drainage of the contrast medium, and disinfection of the endoscopic equipment are expected to reduce the incidence of early-onset cholecystitis. Unlike in the entire group, GB filling with a contrast medium was a risk factor for early-onset cholecystitis in the previous group and was not significant (*p* = 0.056). However, none of the patients in the recent group underwent GB filling with contrast medium, and early-onset cholecystitis was not observed. Therefore, the difference between the previous group and the entire group appears to be due to an insufficient number of patients being included in the study.

Diabetes mellitus has also been found to be a definitive risk factor for early-onset cholecystitis after metal stent placement in the current study. We found that 57.1% (4/7) of the patients with early-onset cholecystitis had diabetes mellitus. According to one study, the risk ratio for hospitalization or physician claims for infectious disease in patients with or without diabetes has been shown to be 1.21 (99% CI 1.20–1.22, *p* < 0.0001) [22]. The result can be explained by diabetic patients being more susceptible to infections. Diabetes was also a risk factor for early-onset cholesterol levels in the previous group. However, diabetes has also been found to be a risk factor for late-onset cholecystitis in the recent group. This may be because the number of patients enrolled was relatively small (14). Further studies are expected to be performed in the future, considering the finding that diabetes is a risk factor for infection.

Tumor invasion of the cystic duct is a known risk factor for cholecystitis after metal stent placement. In the studies conducted by Isayama et al. [23] and Suk et al. [24], cystic duct obstruction was found to be a risk factor for the development of cholecystitis after metal stent placement. However, in the present study, tumor cystic duct invasion alone was not a risk factor for cholecystitis. Tumor invasion of the cystic duct was found to be a risk factor when accompanied by filling the gallbladder with a contrast agent during metal stent placement. These results have also been reported in a previous group. These differences were to be expected for the following reasons. First, both covered and uncovered stents were used in previous studies. In contrast, in this study, all the stents were uncovered, and the effect of covering the narrowed cystic duct orifice was expected to be small. Second, in this study, all Y-stents were placed at the hilum of the bile duct. As the stent position was higher than that used in previous studies, it was expected to cover the cystic duct orifice to a lesser extent. This is believed to be one of the reasons why the length of the common part of the Y-stent in patients with early-onset cholecystitis was significant (*p* = 0.017).

According to the multivariate analysis of the risk factors of early-onset cholecystitis, the length of the common part of the Y-stent was the most important factor (*p* = 0.032; OR, 3.323 (1.111–9.941)). Generally, a long stent is used when the cancer is widespread in the bile ducts. An unfused stent would have relocated widespread cancer masses and affected the GB orifice closure. These observations have also appeared in the previous group. Based on this analysis, early-onset cholecystitis is expected to be reduced by performing the procedure using a Y-stent as early as possible.

Late-onset cholecystitis was observed in 25 (24.0%) patients. Unlike early-onset cholecystitis, the incidence of late-onset cholecystitis is relatively high. We expect that there are several reasons underlying this characteristic. First, in this study, the uncovered Y-configured metal stent was used for biliary drainage, which reduced recurrent cholangitis via adequate drainage. This extended the overall survival period of the patients and thereby increased the number of complications compared with other studies. Second, an endoscopic sphincterotomy was performed in all patients who underwent endoscopic metal stenting. During this process, it is highly probable that food reflux in the duodenum leads to bacterial colonization and subsequent ascending infection.

In the present study, previous early-onset cholecystitis was not a risk factor for late-onset cholecystitis (*p* = 0.771). This may be due to the inability of most patients with early-onset cholecystitis to undergo PTGBD. Two patients treated with antibiotics developed recurrent cholecystitis.

Stent obstruction (*p* = 0.004) and repeat procedures (*p* = 0.01) due to stent malfunction were risk factors for late-onset cholecystitis. These results were similar to those seen in the previous and recent groups. These findings can be attributed to the underlying causes of late-onset cholecystitis, which are believed to involve the progressive obstruction of the cystic duct due to tumor growth, progressive stent occlusion from sludge formation, and the bacterial colonization of bile after endoscopic sphincterotomy. In the multivariate analysis, the reasons for obstruction and the type of repeated procedures were not significant.

Analysis of the previous and recent groups showed some differences from the entire patient group. The recent group had a smaller number of patients compared to the previous group, which was primarily due to the introduction of side-by-side stent procedures for malignant hilar obstruction in more recent times. In addition, unlike in the entire patient group, pre-ERCP cholangitis and GB filling, as opposed to a malignant invasion of the cystic duct, were identified as risk factors in the previous group. However, it is important to note that cholangitis before the procedure and filling the GB with contrast medium during the procedure were less likely to cause cholecystitis after 1 week. It is worth mentioning that these factors were not observed to be risk factors in the recent group. This difference could be attributed to the small number of patients in the group.

Options for cholecystitis management after metal stent placement include antibiotics, percutaneous drainage, and cholecystectomy. Since most patients are unable to undergo surgery, antibiotics and percutaneous gallbladder drainage are generally the treatment of choice for stent-related cholecystitis management [25,26]. In this study, 25 patients underwent percutaneous drainage of the gallbladder. In seven patients, the drainage catheter was removed after 91 ± 35.9 days. However, three patients experienced recurrence, and the drainage catheter had to be reinserted. In other patients, the drainage catheter could not be removed until after their death.

Theoretically, in late-onset cholecystitis, when the cystic duct is completely blocked by tumor masses, there is no distension or secondary infection of the gallbladder after the endoscopic procedure. However, partial obstruction can lead to continuous gallbladder hydrops and recurrent late-onset cholecystitis. Because the Y-stent is a bare stent, it is considered likely that only part of the cystic duct was blocked in most cases. In such cases, the percutaneous drainage catheter cannot be removed from the gallbladder, and drainage must be performed continuously from the gallbladder using a tube, which is extremely uncomfortable for patients. Other drainage techniques, such as endoscopic ultrasound-guided cholecystoduodenostomy and percutaneous or transpapillary cystic duct stenting, should be considered for the patients.

This study had some limitations. First, as this was a retrospective study, there may have been insufficient data available for certain variables. Therefore, caution should be exercised when interpreting the findings. Second, this was a single-center study, resulting in a relatively small number of patients being enrolled, potentially leading to selection bias. However, it is worth highlighting that this study presents more detailed and longer follow-up data for bilateral Y-configured metal stenting in malignant hilar cholangiocarcinoma than other studies.

## 5. Conclusions

The endoscopic insertion of metal stents effectively provides palliative care to patients with malignant biliary obstruction. However, cholecystitis often develops after the procedure. Since cholecystitis affects the patient’s prognosis and quality of life, adequate causation should be exercised, and the patient should be monitored for malignant biliary obstruction. Since early-onset cholecystitis frequently occurs in diabetic patients, it may be helpful to be more careful about GB contrast medium injection during the procedure and to select a shorter length of the Y-stent in the common part. Late-onset cholecystitis is mainly associated with stent obstruction. Thus, careful follow-up may help to determine whether gallstones and cancer progression reduce the function of the stent.

## Figures and Tables

**Table 1:** The characteristics of patients who developed early-onset cholecystitis.

Parameters	Univariable Analysis			Multivariate Analysis		
	Early-Onset Cholecystitis	No Early-Onset Cholecystitis	*p*-Value	Odds Ratio	95% Cl	*p*-Value
No. of patients	7 (6.7)	97 (93.3)				
Gender (M/F)	3 (42.9)/4 (57.1)	52 (53.6)/45 (46.4)	0.582			
Age (year, mean ± SD)	62.7 ± 4.9	72.5 ± 10.8	0.688			
Presence of GB stone	1 (14.3)	13 (13.4)	0.947			
Presence of diabetes	4 (57.1)	22 (22.7)	0.042	4.687	0.782–28.106	0.091
AST	95.3 ± 18.3	89.8 ± 121.32	0.126			
ALT	113.6 ± 33.7	85.4 ± 82.78	0.215			
TB	11.7 ± 8.6	8.9 ± 8.1	0.346			
DB	7.2 ± 5.5	5.4 ± 5.0	0.346			
Pre-ERCP cholangitis	3 (42.9)	23 (23.7)	0.259	1.893	0.311–11.507	0.488
GB filling with contrast medium	5 (71.4)	32 (33.0)	0.040	3.475	0.550–21.974	0.186
Invasion of the cystic duct orifice	6 (85.7)	66 (68.0)	0.074	2.833	0.234–34.308	0.413
GB filling with contrast medium and invasion of the cystic duct	5 (71.4)	18 (18.6)	0.001			
Length of the common part of the Y-stent	5.4 ± 0.8	4.3 ± 0.9	0.017	3.323	1.111–9.941	0.032

Data are presented as mean ± SD. ERCP, endoscopic retrograde cholangiopancreatography; GB, gallbladder; AST, aspartate transaminase; ALT, alanine transaminase; TB, total bilirubin; DB, direct bilirubin.

**Table 2:** The characteristics of patients who developed late-onset cholecystitis.

Parameters	Univariable Analysis			Multivariate Analysis		
	Late-Onset Cholecystitis	No Late-Onset Cholecystitis	*p*-Value	Odds Ratio	95% Cl	*p*-Value
No. of patients	25 (24.0)	79 (76.0)				
Gender (M/F)	10 (40)/15 (60)	45 (57.0)/34 (43.0)	0.139			
Age (year, mean ± SD)	71.4 ± 8.5	72.3 ± 11.5	0.690			
Presence of GB stone	5 (20)	9 (11.4)	0.272	3.295	0.791–13.722	0.101
Presence of diabetes	5 (20.0)	21 (26.6)	0.508	0.707	0.203–2.458	0.586
AST	75.0 ± 60.1	95.8 ± 130.3	0.395			
ALT	98.0 ± 82.2	84.3 ± 79.5	0.533			
TB	6.4 ± 4.9	10.1 ± 8.9	0.418			
DB	4.0 ± 3.2	6.1 ± 5.5	0.418			
Pre-ERCP cholangitis	8 (32.0)	18 (22.8)	0.354	1.295	0.434–3.865	0.643
GB filling with contrast medium	11 (44)	26 (32.9)	0.313	1.135	0.395–3.261	0.814
Invasion of the cystic duct orifice	20 (80)	52 (65.8)	0.219	2.345	0.736–7.474	0.150
GB filling with contrast medium and invasion of the cystic duct	9 (36)	14 (17.7)	0.055			
Length of the common part of the Y-stent	7.3 ± 0.9	7.1 ± 1.1	0.364	1.332	0.786–2.257	0.287
Previous early-onset cholecystitis	2 (8.0)	5 (6.3)	0.771			
Stent patent days	256.4 ± 177.1	115.0 ± 126.1	0.268			
Stent obstruction	21 (84.0)	41 (51.9)	0.004	5.879	1.641–21.059	0.007
- Obstruction reason; ingrowth/overgrowth/ sludge/stone	7 (33.3)/10 (47.6) /3 (14.2)/1 (4.8)	15 (36.6)/19 (46.3) /6 (14.6)/1 (2.4)	0.967			
Repeated procedure due to stent obstruction	21 (84.0)	35 (44.3)	0.001			
- PTBD/ plastic stent/c ERCP/ metal stent/c ERCP	12 (57.1)/7 (33.3)/2 (9.5)	20 (57.1)/12 (34.3)/3 (8.6)	0.992			
Survival days	385.0 ± 245.1	151.7 ± 156.5	0.239			

Data are presented as mean ± SD. ERCP, endoscopic retrograde cholangiopancreatography; GB, gallbladder; AST, aspartate transaminase; ALT, alanine transaminase; TB, total bilirubin; DB, direct bilirubin.

**Table 3:** The characteristics of the previous group of patients (2005–2012) who developed early/late-onset cholecystitis.

Parameters	Early-Onset Cholecystitis	No Early-Onset Cholecystitis	*p*-Value	Late-Onset Cholecystitis	No Late-Onset Cholecystitis	*p*-Value
No. of patients	7 (7.8)	83 (92.2)		24 (26.7)	66 (73.3)	
Gender (M/F)	3 (42.9)/4 (57.1)	46 (55.4)/37 (44.6)	0.522	9 (37.5)/15 (62.5)	39 (59.1)/27 (40.9)	0.179
Age (year, mean ± SD)	62.7 ± 4.9	70.9 ± 10.4	0.530	71.8 ± 8.0	70.1 ± 11.1	0.786
Presence of GB stone	1 (14.3)	12 (14.5)	0.990	5 (20.8)	8 (12.1)	0.204
Presence of diabetes	4 (57.1)	20 (24.1)	0.046	4 (16.7)	19 (28.8)	0.362
AST	95.3 ± 18.3	86.7 ± 114.0	0.105	68.1 ± 58.6	93.6 ± 121.2	0.458
ALT	113.6 ± 33.7	82.1 ± 78.6	0.357	87.1 ± 80.3	83.7 ± 75.7	0.655
TB	11.7 ± 8.6	9.3 ± 8.4	0.392	6.3 ± 4.8	10.5 ± 9.1	0.471
DB	7.2 ± 5.5	5.8 ± 5.3	0.392	4.0 ± 3.1	6.5 ± 5.7	0.471
Pre-ERCP cholangitis	3 (42.9)	15 (18.1)	0.115	8 (33.3)	10 (15.1)	0.027
GB filling with contrast medium	5 (71.4)	29 (34.9)	0.056	11 (45.8)	23 (34.8)	0.174
Invasion of the cystic duct orifice	6 (85.7)	58 (69.9)	0.375	19 (79.2)	45 (68.1)	0.069
GB filling with contrast medium and invasion of the cystic duct	5 (71.4)	16 (19.3)	0.002	9 (37.5)	12 (18.2)	0.025
Length of the common part of the Y-stent (cm)	5.4 ± 0.8	4.4 ± 0.9	0.019	4.6 ± 0.9	4.4 ± 1.0	0.821
Previous early-onset cholecystitis	-	-	-	2 (8.3)	5 (7.5)	0.791
Stent patent days	-	-	-	249.8 ± 197.5	166.0 ± 136.5	0.051
Stent obstruction	-	-	-	20 (24.1)	39 (59.1)	0.004
Repeated procedure due to stent obstruction	-	-	-	20 (24.1)	33 (50)	0.001
Survival days	-	-	-	379.5 ± 261.4	154.4 ± 162.2	0.245

Data are presented as mean ± SD. ERCP, endoscopic retrograde cholangiopancreatography; GB, gallbladder; AST, aspartate transaminase; ALT, alanine transaminase; TB, total bilirubin; DB, direct bilirubin.

**Table 4:** The characteristics of the recent group of patients (2013–2020) who developed late-onset cholecystitis.

Parameters	Late-Onset Cholecystitis	No Late-Onset Cholecystitis	*p*-Value
No. of patients	1 (7.1)	13 (92.9)	
Gender (M/F)	1 (100)/0 (0)	6 (46.2)/7 (53.8)	0.299
Age (year, mean ± SD)	80	80 ± 10.4	0.173
Presence of GB stone	0 (0)	1 (7.7)	0.773
Presence of diabetes	1 (100)	2 (15.4)	0.047
AST	37	124.7 ± 183.68	0.374
ALT	27	71.2 ± 88.37	0.233
TB	0.37	4.8 ± 4.0	0.301
DB	0.21	4.4 ± 3.57	0.301
Pre-ERCP cholangitis	0 (0)	8 (61.5)	0.231
GB filling with contrast medium	0 (0)	3 (23.1)	0.588
Invasion of the cystic duct orifice	1 (100)	7 (53.8)	0.369
GB filling with contrast medium and invasion of the cystic duct	0 (0)	2 (15.4)	0.672
Length of the common part of the Y-stent (cm)	4	4 ± 1.2	0.267
Stent patent days	321	96 ± 128.0	0.082
Stent obstruction	1 (100)	2 (15.4)	0.047
Repeated procedure due to stent obstruction	1 (100)	2 (15.4)	0.047
Survival days	434	179 ± 154.0	0.374

Data are presented as mean ± SD. ERCP, endoscopic retrograde cholangiopancreatography; GB, gallbladder; AST, aspartate transaminase; ALT, alanine transaminase; TB, total bilirubin; DB, direct bilirubin.

## Data Availability

All data used in this study are provided in this article.
